# Polyextremophile engineering: a review of organisms that push the limits of life

**DOI:** 10.3389/fmicb.2024.1341701

**Published:** 2024-06-05

**Authors:** Joaquin Caro-Astorga, Joseph T. Meyerowitz, Devon A. Stork, Una Nattermann, Samantha Piszkiewicz, Lara Vimercati, Petra Schwendner, Antoine Hocher, Charles Cockell, Erika DeBenedictis

**Affiliations:** ^1^The Francis Crick Institute, London, United Kingdom; ^2^Pioneer Research Laboratories, San Francisco, CA, United States; ^3^Pivot Bio, Berkeley, CA, United States; ^4^Department of Ecology and Evolutionary Biology, University of Colorado, Boulder, CO, United States; ^5^University of Florida, Gainesville, FL, United States; ^6^London Institute of Medical Sciences, London, United Kingdom; ^7^UK Centre for Astrobiology, University of Edinburgh, Edinburgh, United Kingdom

**Keywords:** extremophile, directed evolution, functional genomics, ISRU, biomanufacturing

## Abstract

Nature exhibits an enormous diversity of organisms that thrive in extreme environments. From snow algae that reproduce at sub-zero temperatures to radiotrophic fungi that thrive in nuclear radiation at Chernobyl, extreme organisms raise many questions about the limits of life. Is there *any* environment where life could not “find a way”? Although many individual extremophilic organisms have been identified and studied, there remain outstanding questions about the limits of life and the extent to which extreme properties can be enhanced, combined or transferred to new organisms. In this review, we compile the current knowledge on the bioengineering of extremophile microbes. We summarize what is known about the basic mechanisms of extreme adaptations, compile synthetic biology’s efforts to engineer extremophile organisms beyond what is found in nature, and highlight which adaptations can be combined. The basic science of extremophiles can be applied to engineered organisms tailored to specific biomanufacturing needs, such as growth in high temperatures or in the presence of unusual solvents.

## Introduction

Extremophilic microbes have long been studied in hopes of better understanding the origin and limits of life. Extremophile biology is also relevant to biomanufacturing (Ye et al., 2023), where large-scale growth occurs in non-natural, extreme chemical conditions ranging from the use of toxic waste streams as feedstocks to the intentional production of toxic chemicals like butane. The space science community hopes to push the capabilities of biomanufacturing even further for *in situ* resource utilization (ISRU) (Cockell, 2022), especially on human missions to the moon, Mars, and beyond. This will require microbes that are well adapted to chemically unusual feedstocks derived in part from highly oxidized Moon regolith or perchlorate-containing Mars regolith. A microbe that can thrive, growing and metabolizing at high rates, in extreme bioprocessing conditions can enable robust, high-yield, and low-cost synthesis of biological products. We aim to not just understand the basic science of extremophile biology, but also how that basic science supports current and future extremophilic bioengineering.

### Precedent for bioengineering extremophilic traits

Extremophilic tools and traits can be engineered in two ways. Individual extremophilic enzymes such as thermostable amylases, cold-adapted β-glucosidases, DNA-dependent DNA polymerases, and high pH tolerant lipases enable extremophilic catalysis abilities for varied uses (Chien et al., 1976; Zhu et al., 2020). Whole extremophile organisms, including engineered strains, are used to produce ectoine, polyhydroxyalkanoate, polyhydroxybutyrate, and other bioproducts (Tan et al., 2011; Chavan et al., 2021; Hu et al., 2024). Growth in extreme environments also enables new kinds of fermentation, such as non-sterile continuous production in seawater, and gas fermentation in waste gasses from steel production (Yue et al., 2014; Molitor et al., 2016). Biomanufacturing in terrestrial or space processes will require advances in basic fermentation approaches. Desirable extremophilic traits such as tolerance to potentially toxic waste-derived feedstocks, growth in extreme environments hostile to contaminants, and desiccation tolerance open access to entirely new types of bioproduction processes (Chen and Jiang, 2018; Averesch et al., 2023).

The table below summarizes the current understanding of how each category of extremophile adaptation works, and whether there is precedent for deliberately endowing a new organism with this adaptation through bioengineering (Table 1). Growth of microorganisms in some extremes, such as low or high temperature and high radiation, have been comprehensively explored, and are mechanistically quite well understood. Other extremes require more complex equipment to simulate, most notably altered gravity and high and low pressure. This presents a substantial barrier for experimentation. For these extremes, initial studies have been completed, but more research is needed to replicate and interpret results.

**Table 1:** Precedent for bioengineering extremophilic traits.

Environments and applications	Biophysical mechanism of tolerance	Precedent for functional transfer or lab adaptation
Extreme temperatures		
**Low temperatures** 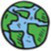 Bioremediation and agricultural use on Earth will see surface temperatures typically from 0°C to 40°C (Daly et al., 2012; Widrlechner et al., 2012). Fermentation at low temperatures is preferred for some food products because it improves retention of flavor volatiles (Liszkowska and Berlowska, 2021). 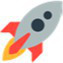 Mars surface temperatures average − 60°C, and hypersaline brines may remain liquid at these temperatures (Gayen et al., 2020; Mapstone et al., 2022). One proposal uses insulation to produce a layer of Mars regolith near 0°C (Wordsworth et al., 2019). Proposed Mars sample return mission architectures require limited heating to preserve evidence of biological processes (Wadhwa et al., 2022).	Adaptations to maintain the fluidity of their membranes and the stability of their proteins at low temperatures, such as cold-active enzymes with high catalytic efficiency at low temperatures, and increased expression of chaperone proteins (D’Amico et al., 2006). Cold shock (Zhang and Gross, 2021) can be sensed and invokes multiple responses.	Growth at 8°C increased 100-fold in *Escherichia coli* from expression of psychrophilic homologs of GroEL/GroES from *Oleispira antarctica* (Ferrer et al., 2003). Expression of chaperone CsHSP17. 5, a heat shock protein from *Castanea sativa* (sweet chestnuts), in *E. coli* improves cell survival after incubation at 4°C for 10 days by 4-fold (Soto et al., 1999). Transferring the gene *hsp17.7* from carrots to *S. cerevisiae* increased cell division rate at low temperature (25°C), achieving stationary phase 3 h faster than wild type (Ko et al., 2017).
**High temperatures** 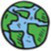 Cooling is a large fixed cost for industrial fermentation, an exothermic process oxidizing sugars and other feedstock into biomass and product. Growth at high temperature reduces cooling requirements and improves contamination tolerance (Abdel-Banat et al., 2010). Growth of organism’s outdoors on Earth may require adaptation to higher temperatures due to climate change. 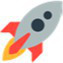 Fermentation in space, on the moon, or on the surface of Mars may require cooling in a vacuum or very low pressure atmosphere, which is typically costly (Von Arx and Delgado, 1991; Tachikawa et al., 2022).	Adaptive mechanisms include: proteome-wide changes improve protein thermostability, synthesis of heat-shock proteins (HSPs) to prevent protein aggregation and assist in protein folding, changes in membrane fluidity, increased supertwists and higher GC content to stabilize DNA, and synthesis of polyamines for RNA protection (Terui et al., 2005; Nakashima et al., 2017; Kobayashi et al., 2022). Heat shock (Roncarati and Scarlato, 2017) can be sensed and invokes multiple responses.	Over-expression of transcriptional regulator evgA in *E. coli* enabled growth at 50°C (Christ and Chin, 2008). Transfer of the cold shock gene *cspL* from *Bacillus coagulans* improves growth at high temperatures when expressed in *E. coli* (2-fold improvement at 45°C) and *S. cerevisiae* (2-fold improvement at 36°C) (Zhou et al., 2021). Expression of chaperone *CsHSP17.5*, a heat shock gene from *C. sativa* (sweet chestnuts), in *E. coli* improves cell survival after 50°C heat shock by 2-fold (Soto et al., 1999). *S. cerevisiae* was evolved to thrive at 42°C, resulting in over 100 genomic mutations (Huang et al., 2018). Passaging at increasing temperature was used to evolve the diatom *Nitzschia inconspicua* for growth at 37.5°C, 2°C above the wild-type limit, though the genetic changes were not studied (LaPanse et al., 2023).
**Freeze thaw** 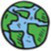 Long term storage of biomaterials, such as seed cultures of probiotics or research strains, must tolerate freeze–thaw (Kwon et al., 2018). Use of microbe’s outdoors may require tolerance to freeze thawing, depending on the season. 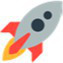 Growth in certain less controlled environments, such as incompletely insulated Mars surface exposed to a diurnal cycle, will require surviving repeated freeze–thaw cycles (Wordsworth et al., 2019).	Some organisms can tolerate daily freeze–thaw (Vimercati et al., 2016; Zhang et al., 2020) but the underlying mechanism for freeze–thaw tolerance is not well understood.	*Lactobacillus rhamnosus* was evolved for 150 cycles to better tolerate freeze–thaw, resulting in a 50% improvement in freeze thaw survival (Kwon et al., 2018). Industrial baker’s yeast was UV mutagenized and evolved for 200 freeze–thaw cycles, resulting in a strain, AT25, that exhibits improved tolerance to freeze thaw stress (Teunissen et al., 2002). Overexpression of aquaporins for water efflux in AT25 further increased freeze–thaw survival up to ten-fold (Tanghe et al., 2002).
Extreme physicochemical environments		
**High UV or ionizing radiation** 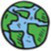 Bioremediation of radionuclides such as uranium can rely on absorption or accumulation in cells or redox/precipitation reactions to reduce metal ion migration in groundwater (Newsome et al., 2014). 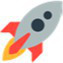 High ionizing radiation may be beneficial for biomass farming with minimal radiation shielding on Mars (Patel et al., 2004).	Adaptive mechanisms include: polyploidy, expression of anti-oxidative proteins, and expression of DNA stress damage response proteins.	Radiation survival in *E. coli* was improved by more than two orders of magnitude by the expression of Dsup protein from tardigrade *Ramazzottius varieornatus* that binds to and protects DNA from X-ray damage (Chavez et al., 2019; Puig et al., 2021). Surprisingly, Dsup was also transferred to yeast, protecting yeast from oxidative damage, but increasing sensitivity to UV (Aguilar et al., 2023). Integration of the tyrosinase gene *Tyr* from *Aspergillus fumigatus* into the entomopathogen *Beauveria bassiana* increasing its conidium resistance to UV-B radiation 0.3 J/cm^2^ for the WT to 0.5 J/cm^2^ (Shang et al., 2012).
**High oxidizer concentration** 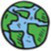 Contamination sites on earth can accumulate significant perchlorate. In one example, areas under industrial waste ponds accumulated up to 3% perchlorate, and bioremediation removed perchlorate from the surrounding groundwater (Levakov et al., 2019). 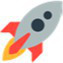 Mars soil contains high perchlorate, at one site around 0.6% (Hecht et al., 2009) posing challenges for biological ISRU. Some cyanobacteria have been shown to tolerate ClO_4_^−^ at concentration up to 1% (Rzymski et al., 2022), and one psychrophile can tolerate up to 10% ClO_4_^−^ at low temperature (Heinz et al., 2019).	Adaptive mechanisms include: high osmotic pressure affecting protein composition and synthesis of membrane transporters or metabolites to deal with extreme environmental parameters (Vera-Bernal and Martínez-Espinosa, 2021).	Expression in *E. coli* of genes from metagenomic DNA sourced from hyper-saline environments can improve survival on 125 mM sodium perchlorate by three orders of magnitude (Díaz-Rullo et al., 2021). The metagenomic genes of interest included genes of unknown function as well as genes involved in DNA protection and repair.
**High salinity** 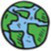 New bioprocessing techniques include non-sterile continuous processes in sea water (Tan et al., 2011; Yue et al., 2014). Stressed microbial survival has been measured at water activity of 0.6 (Bolhuis et al., 2006), and optimal growth can occur at salinities as high as 30% w/v (Corral et al., 2019). 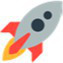 Martian hypersaline brines may exceed 25% salt by weight, with water activities of 0.76 or lower (Gekas et al., 1998; Fox-Powell et al., 2016). The ocean of Europa provides remote signatures of salt water of unknown salinity (Hand and Chyba, 2007; Trumbo et al., 2019).	Salt tolerance can be achieved by expressing pumps or osmotic pressure regulators. It can also be tolerated by producing sugars, alcohols in order to restore osmotic pressure. Halophilic microorganisms achieve osmotic balance through two fundamentally different strategies: salt-in, where outside salts are balanced with high intracellular salt concentrations, using K+ rather than Na + as the main cation in the cytoplasm, or salt-out, by excluding salts from the cytoplasm and accumulating small, generally uncharged or zwitterionic, organic compounds as ‘compatible’ solutes (Sévin et al., 2016; Kumar et al., 2020). Another mechanism is proteome evolution. High concentration of salts dehydrate proteins and induce their aggregation. Some organisms protect their proteins by displaying acidic residues that retain water around the protein, preventing its dehydration (DasSarma and DasSarma, 2015; Cabello-Yeves and Rodriguez-Valera, 2019).	Enhancing salt tolerance is perhaps the most frequently studied extremophile bioengineering task in the literature to date (Kumar et al., 2020). Salt tolerance can be enhanced through transfer of genes from salt-tolerant species: IrrE gene from *Deinococcus radiodurans* increase survival of *E. coli* by 3 orders of magnitude to 0.65 M NaCl for 2 h (Pan et al., 2009); the gene Ds-26-16 from *Dunalliela* expressed in *E. coli* increased survival to 500 mM NaCl by 50% (Wang et al., 2016); introduction of *DnaK* from *Bacillus halodurans* into *E. coli* increased survival in 400 mM NaCl by 4-fold (Vahdani et al., 2019). Recombinant *S. cerevisiae* expressing the gene *pprI* of *D. radiodurans* grew well in 1.2 M NaCl (7%), while normal strain could only survive under 0.85 M (Hossein Helalat et al., 2019). The cyanobacteria *Synechococcus elongatus* engineered to express the ectoine biosynthetic pathway increased by 80% its ability to grow in 400 mM NaCl (Dong et al., 2023). Choline oxidase A (*codA*) from *Arthrobacter globiformis* produced increased glycinebetaine solutes in the microalga *Chlamydomonas reinhardtii*, improving growth during salt stress (Hema et al., 2007).
**Extreme pH** 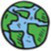 Some techniques, including dye production, metal remediation in alkaline soils, and microbial fuel cell electric production require growth at high pH (Preiss et al., 2015). Other processes in food manufacture, organic waste recycling, and mineral bioleaching are performed at low pH (Atasoy et al., 2024; Sun et al., 2024). 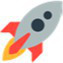 One basaltic regolith simulant was unable to grow plant life due to a pH > 9.0, suggesting some regions of Mars will present pH-related challenges for microbial growth (Eichler et al., 2021).	With few exceptions, these organisms maintain a cellular pH near neutral. Reducing the entry of protons into the cells by having less permeable membranes, modulating the size of membrane channels, generating a chemioosmotic gradient via potassium ATPases, pumping excess protons out from the cytoplasm through proton pumps, and maintaining the integrity and fluidity of cell membranes by modulating fatty acid composition (Guan and Liu, 2020). Non-neutral intracellular pH requires extensive adaptation both at low pH (Menzel and Gottschalk, 1985; Mullins et al., 2012) and at high pH (Sturr et al., 1994; Janto et al., 2011).	Genes of unknown function can be isolated from metagenomic DNA sources from acidic environments using functional genomics in *E. coli* (Guazzaroni et al., 2013). Several of these genes improve survival after temporary exposure to acidic conditions by up to seven orders of magnitude when expressed recombinantly in *E. coli*, *Pseudomonas putida, and B. subtilis* (Guazzaroni et al., 2013). The expression of the halophilic heat shock gene *DnaK* from *B. halodurans* increased *E. coli* survival by 2.5 folds at pH 9.5 (Vahdani et al., 2019).
**High heavy metal concentration** 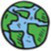 In heavy metal contaminated sites, microbes can sequester soluble metal ions or turn them into less toxic forms (Sun et al., 2024). 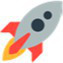 Mars regolith is estimated to contain 0.3% chromic oxide (West et al., 1999), and potentially hazardous levels under 150 ppm of hexavalent chromium, arsenic, and cadmium (National Research Council, Division on Engineering and Physical Sciences, 2002).	Preventing metals from entering the cell by binding them to the external surface, efflux transporters that excrete toxic metals from the cell, and sequestering metals into stable internal inclusion bodies, production of metal-binding compounds (Gall et al., 2015), known efflux pumps and metal ion preferences (Collard et al., 1994).	Long-term evolution of *Shewanella oneidensis* can improve tolerance to toxic levels of 190 mg/L of Cr(VI) (Xiao et al., 2019). Heterologous expression of phytochelatin synthase from *Pyrus calleryana* in *E. coli* allows growth in up to 2.0 mM Cd^2+^, 4.0 mM Cu^2+^, or 200 μ MHg^2+^ (Li et al., 2015). *S. cerevisiae* bioengineered to express on its surface C-terminal half of alpha-agglutinin fused to 6xHis was four times more resistant to copper (up to 4 mM) than the parent (below 1 mM) (Kuroda et al., 2001). Expression of the metallothionein-like (MT-like) gene from *Festuca rubra* in the micro algae *Chlamydomonas reinhardtii* chloroplast genome increased IC50 to Cd^2+^ by 55% (Han et al., 2008).
**High organic solvent concentration** 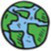 Fermentation of biofuels such as isobutanol or ethanol requires microbes tolerant of high product titer (Sardessai and Bhosle, 2004; Dunlop et al., 2011; Varize et al., 2022). 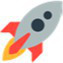 Bioproduction of rocket propellant on Mars and other processes may require solvent extractions and *in situ* solvent production (Syu, 2001; Kruyer et al., 2021).	Adaptive mechanisms include: modifying the fatty acid composition of the cell membrane to maintain optimal fluidity, increasing the level of cyclopropane fatty acids (CFAs), and adjusting the polar head groups of phospholipids (Schalck et al., 2021).	Laboratory evolution of *E. coli* to grow optimally achieved tolerance at 60–400% higher concentrations than initial toxic levels in the presence of 11 industrial chemicals (1,2-propanediol, 2,3-butanediol, glutarate, adipate, putrescine, hexamethylenediamine, butanol, isobutyrate, coumarate, octanoate, hexanoate) (Lennen et al., 2023). In a different study, the transfer of efflux pump genes to *E. coli* also showed 30–300% improves in tolerance to multiple industrial molecules related to biofuel industry (Dunlop et al., 2011).
**High microbial growth inhibitor concentration** 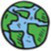 Widely used low-cost feedstocks for fermentation typically contain high concentrations of plant derived reactive lignin monomers and dehydration products of sugars (Klinke et al., 2004; Devi et al., 2021; Jayakody and Jin, 2021). Environmentally persistent toxins such as dioxins can be degraded biologically (Kearney et al., 1972; Nhung et al., 2022). 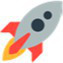 Plants grown on Mars can support similar fermentation processes, with the same issues related to toxicity of less purified feedstock (Duri et al., 2022). Examples of inhibitor molecules include: *Aldehydes*: these chemically reactive aldehydes can obstruct the synthesis of specific compounds (Jayakody and Jin, 2021). Furfural and HMF are typical examples of metabolites produced from lignocellulose degradation (Liu, 2021). *Dioxins* are mutagenic molecules that induce oxidative stress in eukaryotes and prokaryotes (Min et al., 2003; Reichard et al., 2006). *Phenolic compounds* like vanillin are toxic to industrial yeast (Fletcher and Baetz, 2020; Liu et al., 2021).	Mechanisms for tolerance to toxic chemicals include degradation, efflux pumps, or mutagenesis of the affected target. Several mechanisms have been identified with potential transferability to create resistant strains, for example to phenolic compounds produced during lignocellulose degradation (Fletcher and Baetz, 2020).	Modifying the native Adh1p enzyme in *S. cerevisiae* by replacing a single amino acid (Y295C) altered its substrate preference from a short-chain aldehyde to furfural reduction (Laadan et al., 2014). In *S. cerevisiae*, deletion of the gene *YRR1 produces 9-fold* higher OD in cultures with 6 mM vanillin (Wang et al., 2017). Deletion of *BNA7* increases OD by 50% in liquid cultures with 10 mM ferulic acid (Fletcher et al., 2019). The bialaphos resistance (*BAR*) gene from *Streptomyces* spp. have been widely transferred to crops to resist herbicides and also used as a selectable marker in *Cercospora kikuchii* fungi (Upchurch et al., 1994).
**Low water activity and desiccation** 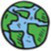 Desiccation allows for non-cold chain storage and transport of microbial strains for research (Prakash et al., 2013), probiotics, and microbial transplants (Haifer et al., 2021). Bioremediation of polycyclic aromatic hydrocarbons has been tested in areas with seasonal drought (Vilchez and Manzanera, 2011). 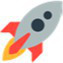 Water activity on Mars is expected to be lower now than the low levels estimated for early Mars (Tosca et al., 2008), and slope streaks suggest transient surface flows of liquid brine (Bhardwaj et al., 2017). Lower Venus atmosphere may sustain desiccation-tolerant spores and sporadic growth in droplets (Seager et al., 2021).	Adaptive mechanisms include: changes in membrane lipid composition (Haque and Russell, 2004), and accumulation of chaotropic metabolites (Chin et al., 2010; Cray et al., 2013, 2015).	The recombinant expression of sucrose-6-phosphate synthase from the cyanobacterium *Synechocystis* to *E. coli* increased bacteria survival to desiccation by 10,000 fold (Billi et al., 2000).
**Extreme pressure or gravity** 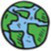 Microbial growth in oil wells includes culturable strains preferring growth at 20 MPa (Roumagnac et al., 2020). Increased pressure may improve certain gas fermentation processes (Van Hecke et al., 2019). Separative processes such as hydrocyclones and ultrafiltration maintain cell viability while removing product from fermentation (Ferras et al., 1986; Bicalho et al., 2012). While atmospheric bacteria have been detected in the upper troposphere at 15 km altitude and 0.01 MPa, it is unclear if growth occurs at this altitude and pressure (DeLeon-Rodriguez et al., 2013). 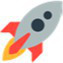 Oceans under Europa’s ice surface can reach 20–200+ MPa (Naganuma and Uematsu, 1998; Howell, 2020), layers of Venus atmosphere with life-compatible temperatures can reach 0.2 MPa (Seager et al., 2021), and the surface of Mars has a low atmospheric pressure near 600 Pa (Banfield et al., 2020), providing a full range of possible environmental pressures for growth. Space environments such as space stations, asteroids, and the moon provide varied low gravity challenges for *in situ* resource utilization (Cockell, 2022), and exoplanets in habitable zones can reach 36 Earth masses (PHL @ UPR Arecibo - habitable worlds catalog, n.d.). Low gas pressures have been shown to support algae and cyanobacterial growth for ISRU (Cycil et al., 2021; Verseux et al., 2021; Gumulya et al., 2022).	The mechanism by which organisms tolerate high or low pressure is largely unknown (Schwendner and Schuerger, 2020). Prokaryotes including *E. coli* and *Paracoccus denitrificans* have been shown to proliferate at up to 400,000 x g (Deguchi et al., 2011). Low gravity results in lack of convection, which can cause waste buildup, nutrient starvation, and issues with respiration (Musgrave et al., 1997; Monje et al., 2020). The effects of microgravity on microbial growth are still under debate (Huang et al., 2018), but have been studied (Zea et al., 2016, 2017). There is no universal bacterial response to microgravity (Sharma and Curtis, 2022).	Heterologous expression of a piezotolerant protein has been demonstrated (Kasahara et al., 2009), but improved whole-cell tolerance to high pressure has not. Adaptive evolution has been used to adapt *B. subtilis* to grow in liquid culture that is aerated with low-pressure air (Nicholson et al., 2010), although this may not be representative of low pressure growth in non-liquid culture.
**Low essential elements** 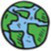 Added phosphate may be required for fermentation, depending on feedstock (Scherer et al., 2009; Van Dijk et al., 2020), and has historically been derived from non-renewable resources such as guano deposits and rare surface deposits of phosphate rock (Van Vuuren et al., 2010; Cordell and White, 2014). Microbial methods can solubilize phosphate for growth (Jones and Oburger, 2011), and microbial sensors for phosphate limitation have been engineered (Larsson et al., 2024). 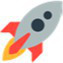 Mars appears to contain the essential elements for microbial growth (Klingler et al., 1989; Kasiviswanathan et al., 2022). Other environments such as Europa, Enceladus, or Titan have uncertain environments that may be deficient in essential components for life (Hoffman et al., 1979; Hendrix et al., 2019).	In absence of fixed nitrogen, nitrogenase expression (Takimoto et al., 2022). In absence of phosphate, increased phosphatase and ribonuclease activity. In absence of sulfur, increased transporter and arylsulfatase expression (Irihimovitch and Yehudai-Resheff, 2008). In absence of iron, siderophore production (Boiteau et al., 2016). Adaptations to low magnesium have been observed but the mechanisms are not well understood (Tindall et al., 1980; Soliman and Trüper, 1982).	Improved growth in phosphate limiting conditions shown with engineered phosphate solubilization (Wagh et al., 2014). Heterologous expression of genes for nitrogen fixation (Tatemichi et al., 2021) and associated co-factor(s) (Solomon et al., 2020), sulfate scavenging, and siderophore production (Puja et al., 2023) has been demonstrated, but improved whole-cell tolerances to the associated stressors have not.

A compilation of extremophile properties, known mechanisms of adaptation, and precedent for bioengineering this property in a new organism. These extremes are relevant in industrial biomanufacturing terrestrially (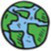), and/or in biomanufacturing in space science applications (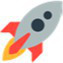).

Low gravity is an especially challenging extreme to study due to the cost and technical complexity of conducting experiments. Low gravity eliminates convection, which may substantially alter the function of microorganisms in ways that may be difficult to simulate on the ground. Low gravity simulation devices can prevent sedimentation but do not eliminate convection (Vroom et al., 2022), and it remains unknown the extent to which these devices are a good proxy for low gravity microbial growth. To reliably investigate low gravity conditions, experiments must be done on experimental platforms in space, such as in Low Earth Orbit.

Low atmospheric pressure would similarly benefit from further research. One paper adapts *Bacillus subtilis* to grow better in liquid culture exposed to low-pressure atmospheric conditions (Nicholson et al., 2010). However, it is unclear whether the experimental setup for this study selects for ‘growth at low pressure’ or simply growth in less well-aerated media. Analysis of the accumulated mutations suggests evolution was driven by the particular experimental conditions rather than low pressure. Mutations in *rnjB*, an RNAse, improves growth at 27°C regardless of air pressure (Waters et al., 2015), and can be interpreted as an adaptation of *B. subtilis* for low temperature. The *rnjB* mutation could account for most of the fitness gain, although additional mutations were also observed (Waters et al., 2021) impacting membrane fluidity and the regulation of anaerobic metabolism such as increased expression of nitrate reductases. Studies of organisms growing on solid media under low pressure may clarify some of these issues.

Tolerance to extremes can be engineered by directly transferring specific genes that improve microbes performance. For example, the transfer of the carrot gene *hps17.7* to *Saccharomyces cerevisiae* improved both growth rate and maximum culture density under low temperature (25°C) and acidic conditions (pH 4) plus high osmolarity (0.8 M sorbitol). Percentage of survival at 47°C increased from 15% of the wild type to 38% of the engineered strain (Ko et al., 2017). This is one of the examples where genetic parts from one species can improve survival for another.

### Thrive versus survive

**Figure 1:**
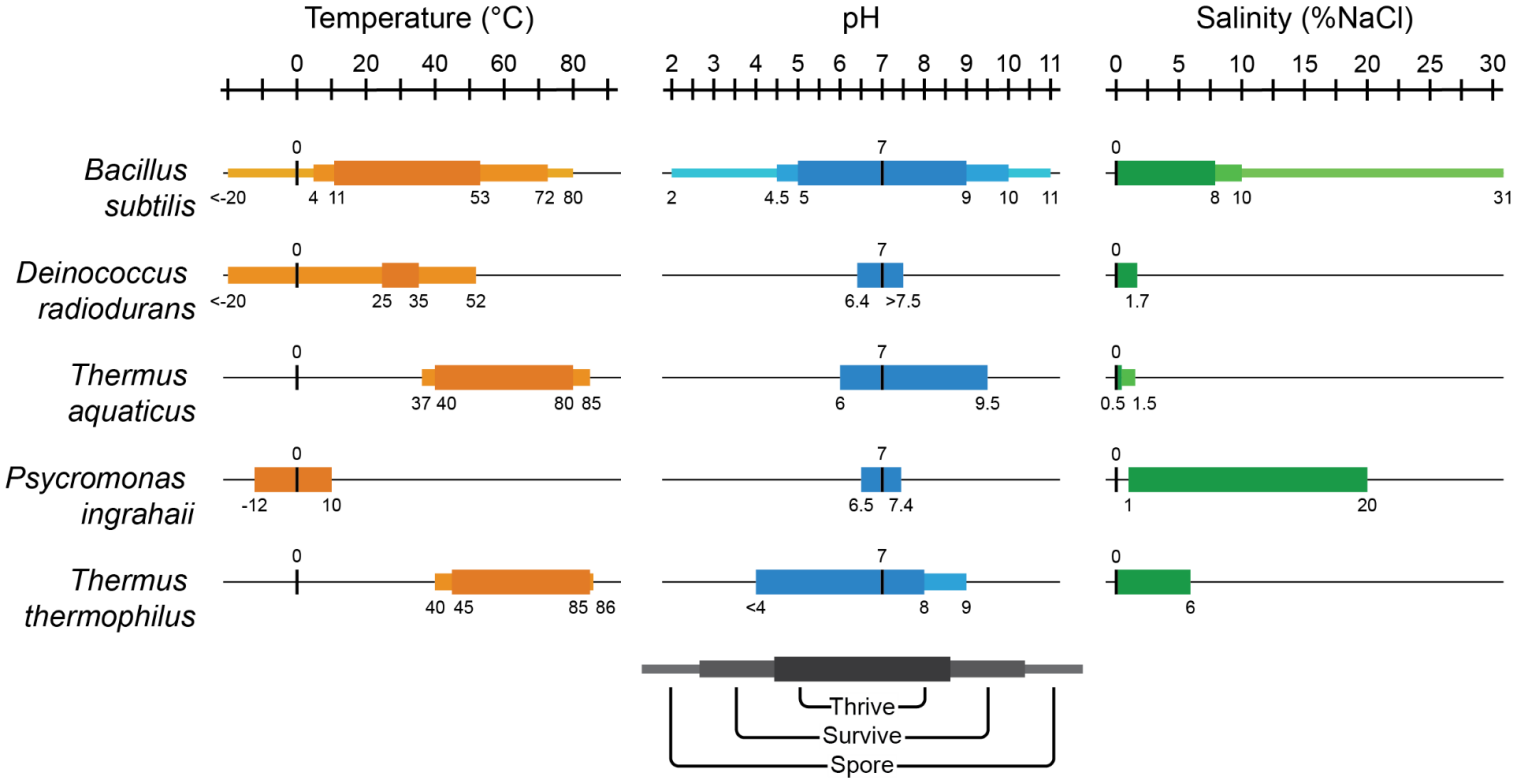
Microbes can survive conditions too extreme for growth. A visualization of the thriving and survival limits of organisms. For a list of citations for each organism in each condition, see Table S1. Graphs indicate the limits of thriving and survival in extreme conditions of temperature, pH, and salinity (% NaCl) for each of the five organisms.

When we envision using extremophiles for biomanufacturing, we require microbes that are capable of rapidly producing biomass under extreme conditions, not just surviving in a dormant state. The literature often conflates the ability to survive temporary exposure to extreme conditions with the ability to thrive and reproduce. Here, we explore the known limits of five well-studied extremophilic organisms; *Bacillus subtilis, Deinococcus radiodurans*, *Thermus aquaticus*, *Psycromonas ingrahaii*, and *Thermus thermophilus*; and their ability to thrive and survive in extreme environments (Figure 1, references in Supplementary Table S1). Engineered extremophiles should be measured against two separate benchmarks: their ability to thrive in an extreme environment (divide under extreme conditions) and separately their ability to survive (tolerate and reproduce *after* temporary exposure to even more extreme conditions). A specific example of this distinction can be seen between related alkalophilic and alkaline-tolerant *Bacillus* spp. (Guffanti et al., 1980). These strains differ in pH homeostatic mechanisms and growth pH range, though their cytoplasmic pH ranges share a common alkaline limit.

Some organisms are capable of entering dormant states, becoming unable to replicate, but even more capable of surviving extreme conditions. For example, *Bacillus* experiences sporulation in which a copy of the genome to be encased in a metabolically inactive and well protected desiccated spore. When favorable conditions return, the spore can germinate, shedding its protective layers and initiating the formation of a new vegetative cell through the reactivation of essential cellular processes (McKenney et al., 2013). Organisms when in sporulated form can survive even more extreme conditions than their non-supported equivalents (Cho and Chung, 2020).

### Somatic adaption versus genetic mutation

The extremophile literature often focuses on genome-level changes as the primary drivers of extremophilic properties. However, simply modulating the expression of endogenous genes is a more subtle yet equally profound aspect of the survival strategy of extremophiles. The dynamic nature of gene expression allows organisms to swiftly react to their environment, mounting an immediate defense against stressors without the long-term commitments tied to genetic mutations or new gene acquisition. For example, heavy water (D_2_O) stress in a variety of organisms is most consistent with adaptation being driven by changes in gene expression, rather than by genomic mutations (Katz and Crespi, 1966), and heterologous expression of a *Deinococcus radiodurans* transcriptional regulator alone can improve varied stress tolerances in multiple species (Wang et al., 2020). Extremophile bioengineering studies should separately characterize the contribution of gene reregulation, and the contribution of genome-level changes.

### The root cause of stress in extreme conditions

**Figure 2:**
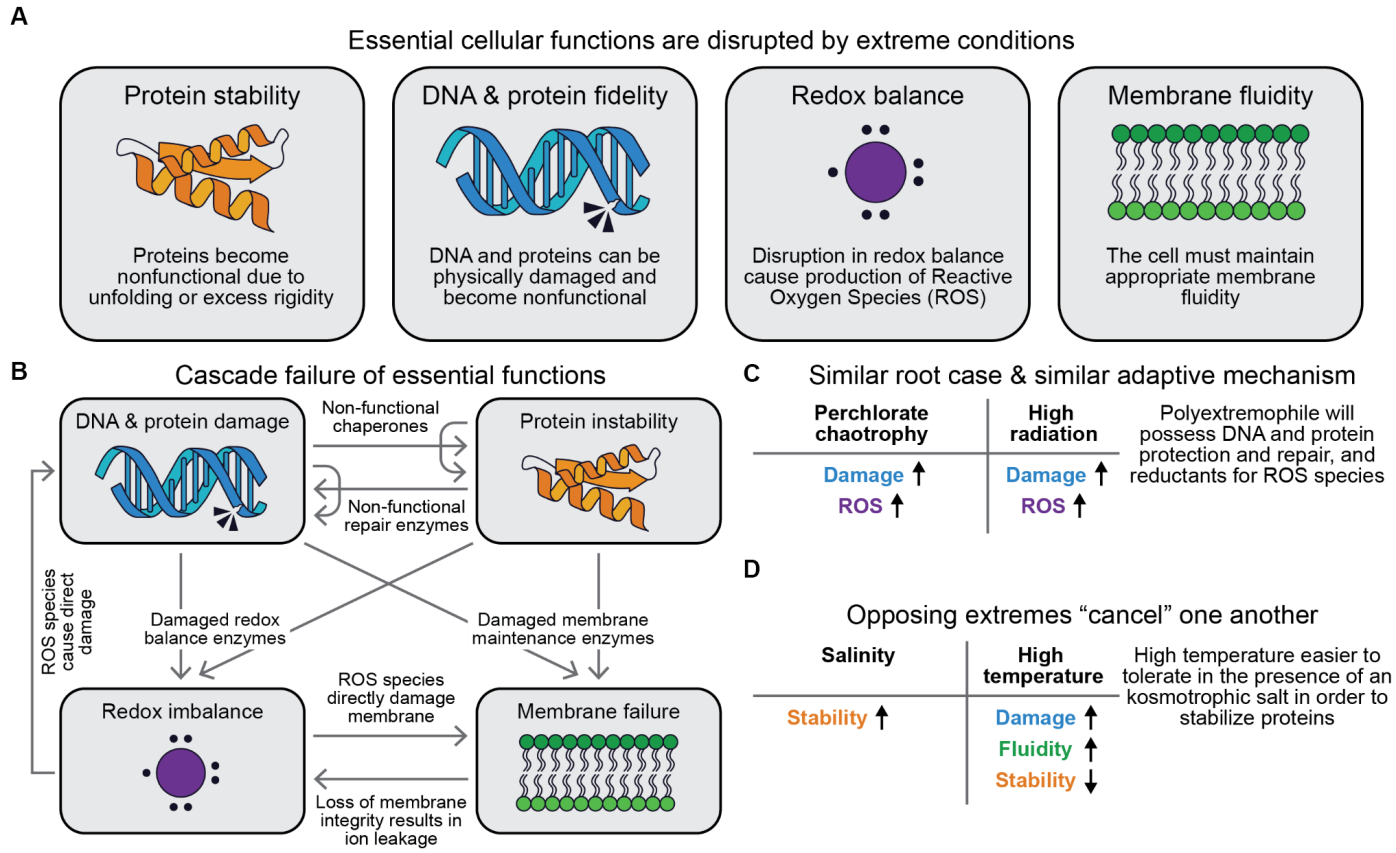
Root causes of stress under extreme conditions. There are several essential cellular functions that are frequently the proximal cause of disruption in extreme conditions **(A)**. When an essential function is disrupted, it can lead to disruption of other essential functions, resulting in a cascade of failure **(B)**. Resistance mechanisms can protect against multiple extremes with the same root cause **(C)**, and stressors with opposite root causes can be easier to tolerate together than separately **(D)**.

In order to flourish, the cell must perform many essential functions, such as maintaining appropriate redox balance, membrane fluidity, protein stability balance, and limiting damage to DNA and proteins (Figure 2A). Studies of model microbes like *E. coli* and *B. subtilus* as well as non-model microbes like *Lactobacillus delbrueckii* and *Acidithiobacillus ferrooxidans* have begun to isolate the proximal mechanism by which extreme conditions disrupt these essential functions (Table 2). There are four broad classes of proximal cases of stress in extreme conditions: generation of reactive oxygen species (ROS), damaging DNA or proteins in ways that break or form chemical bonds, destabilizing the fold of a protein in ways that do not break chemical bonds, and altering the fluidity of the cell membrane. Different stressors most directly impact one or several of these proximal causes of stress. For example, high temperature directly destabilizes folded proteins and increases the error rate during DNA replication (Velichko et al., 2012).

**Table 2:** Direct effects of extreme conditions.

	Reactive oxygen species (ROS)	DNA and protein damage	Impact on protein stability	Impact on membrane fluidity See review (Mykytczuk et al., 2007).
High temperature Damage↑ Stability↓ Fluidity↑	–	DNA replication at high temperature is more error prone (Xue et al., 2021).	High temperature can unfold proteins, decreasing stability. **This effect is pronounced and well-studied.**	High temperature increases membrane fluidity and low temperature decreases membrane fluidity (Mykytczuk et al., 2007). **This effect is pronounced and well-studied.**
Desiccation Stability↓ Fluidity↓	Disrupted respiration and impaired oxidative stress response cause ROS accumulation during desiccation (França et al., 2007), and in multiple species increased catalase expression is seen during xerostress (Scales et al., 2023).	Both DNA (Lebre et al., 2017) and proteins (Laskowska and Kuczyńska-Wiśnik, 2020) are damaged during desiccation by mechanisms aside from ROS such as DNA alkylation and Mallliard reaction protein cross-linking (Billi and Potts, 2002).	Lack of hydration shell results in misfolding and aggregation of proteins (Greffe and Michiels, 2020).	The reduced volume of dehydrated cells results in the concentration of the membrane. There is some evidence that growth in dehydrating conditions, such as in glycerol, decreases membrane fluidity (Beney et al., 2004; Tymczyszyn et al., 2005).
High Radiation ROS↑ Damage↑	Ionizing radiation can directly generate ROS. **This effect is pronounced and well-studied.**	Ionizing radiation can directly damage DNA or proteins. **This effect is pronounced and well-studied.**	–	–
High acidity Damage↑ Stability↓ Fluidity↓	–	Acidic conditions can directly damage DNA through depurination (An et al., 2014).	Acidic conditions can denature proteins by disrupting hydrogen bonding and electrostatics.	Influx of protons is prevented by decreasing membrane fluidity (Mykytczuk et al., 2010).
High pressure Stability↓	–	High pressure is not thought to directly cause damage to DNA and proteins.	High pressure decreases protein stability (Williamson and Kitahara, 2019).	High pressure may physically damage the cell membrane, but it is unclear whether or not there is a consistent, direct impact on membrane fluidity (Pokorny et al., 2005; Mykytczuk et al., 2007).
Salinity induced osmotic pressure Stability↓ or ↑	–	–	Ions impact the behavior of protein stability, folding, and aggregation. Different salts can increase or decrease stability and folding, use the Hofmeister series as a reference (Zhang and Cremer, 2006) and also consider chaotrophicity (Cray et al., 2013). **This effect is pronounced and well-studied.**	Salts can impact membrane fluidity (Kellermann et al., 2016).
Perchlorate (ClO_4_^−^) chaotropy ROS↑ Damage↑ Stability↓ Fluidity↓	Perchlorate generates ROS both directly, and indirectly by disrupting proteins that maintain cellular redox balance.	Perchlorate can denature proteins and precipitate nucleic acids through its chaotropy. It also induces oxidative stress that can damage DNA and proteins.	Perchlorate is highly chaotropic and thus destabilizes protein folds.	Perchlorate is highly chaotropic and thus disrupts lipid membranes.

Extreme conditions (rows) and their direct effects on essential cellular processes (columns).

In addition to direct disruption of important cellular functions, breakdown of one function can lead to a further cascade of failures, such as unfolded DNA repair proteins further increasing the effective DNA replication error rate (Figure 2B). Understanding the root cause of stress in extreme conditions can allow us to reason about which polyextremophiles are biophysically realistic, and which adaptations they might possess. Organisms can tolerate multiple extremes at once if those extremes have the same root cause and thus a similar tolerance mechanism (Figure 2C), for example, the same genes enhance tolerance to both perchlorate and UV radiation in *E. coli* (Lamprecht-Grandío et al., 2020). For example: perchlorate tolerance and high radiation tolerance are both enabled by improved DNA and protein protection and repair (Slade and Radman, 2011; Lamprecht-Grandío et al., 2020). Freezing environment threats comprise crystals formation and local/temporal solute concentration. Similarly, salt tolerance and freeze–thaw tolerance can be synergistic: a metagenomic study of organisms from brines and alkaline lakes found that these salt-tolerant microbes are also 1,000-fold more resistant to freeze–thaw due to high intracellular levels of osmolytes and biofilm formation (Wilson et al., 2012). Conversely, some combinations of extremes are easier to tolerate together than separately because they exert opposing root causes (Figure 2D). High temperature is easier to tolerate in the presence of kosmotropic salts such as NaCl (Chin et al., 2010). Low temperature is easier to tolerate in the presence of chaotropic salts such as MgCl_2_ (Hallsworth et al., 2007; Chin et al., 2010). Haloalkaliphiles demonstrate adaptations to combined stresses (Wiegel and Kevbrin, 2004; Mesbah and Wiegel, 2012) and apparent trade-offs between adaptation to each separate stress (Mesbah and Wiegel, 2011; Banciu and Muntyan, 2015).

However, more research is needed into the mechanism of action of different stress adaptations. Not all extremophile organisms exhibit correlation between growth in multiple extremes that would be expected from Table 2. Early studies showed the *D. radiodurans* stress response to ionizing radiation shares mechanisms with desiccation response, suggesting adaptation to desiccation causes radiation tolerance (Shukla et al., 2007; Ujaoney et al., 2017). Later studies show desiccation stress and radiation tolerance are not correlated in anaerobes, while presence of certain manganese complexes was predictive of radiation tolerance across bacteria, fungi, archaea, and eukarya (Daly, 2009; Sharma et al., 2017; Beblo-Vranesevic et al., 2018). It is unknown how many different adaptations may be used to cope with a particular combination of extremes, if any. For example, there are no known microbes that grow robustly at extreme high and low pH (Jin and Kirk, 2018) or at extreme high and low temperature (Wiegel, 1990), though some organisms are claimed to tolerate ranges of 10 pH units and 60°C (Pandey et al., 2014). This may demonstrate a fundamental limit for microbial metabolism, or it may be a reflection of evolution in environments with limited variation in pH and temperature. Knowledge of compatibility, trade-offs, and relative efficiency between known adaptations will improve design and bioengineering of microbes with extremophilic traits.

### Approaches for polyextremophile bioengineering

There are several approaches to engineer or enhance extreme properties in microbes depending on the type of stress to be addressed and the amount of prior knowledge about tolerance mechanisms. Biocontainment is an overarching consideration when engaging with an extremophile engineering campaign to prevent the release and uncontrolled spread of genetically engineered organisms. Rational design requires adding exogenous DNA with a known function, such as the tardigrade *Dsup* DNA repair gene which enhances survival by 2 orders of magnitude when transferred to *E. coli* grown in harsh conditions (Puig et al., 2021). Knowledge of specific extremophilic genes like tardigrade *Dsup*, as well as genome-scale models such as flux balance analysis (Noirungsee et al., 2024; Saldivar et al., 2024), can both be invaluable for engineering (Swayambhu et al., 2020). If there are no known genes with the needed function, metagenomic libraries sourced from extreme environments can search for genes that confer protection without foreknowledge of the sequence-function relationships (Biver et al., 2014; Culligan et al., 2014; Forsberg et al., 2016; Ausec et al., 2017).

With or without rational genetic engineering, directed evolution can be applied to improve desired properties. In directed evolution, where a library of variants from a starting organism are made, fitness is measured, and improved variants are used as the starting point for iterative rounds of improvement. In cases where an organism’s ability to grow in new extreme conditions is the evolutionary goal, the process is called adaptive lab evolution (Mavrommati et al., 2022). Changes across the whole proteome, not just a single protein or pathway, are required for global adaptations to multi-target stresses (Fernandes et al., 2023) such as high temperature (Deatherage et al., 2017), high salinity (Dhar et al., 2011), and high oxidative stress (Papiran and Hamedi, 2021). Some metabolic adaptations such as improved growth on alternative carbon sources (Chen et al., 2020; Espinosa et al., 2020) or efficient photosynthesis in high light (Dann et al., 2021) have been demonstrated, each requiring tens to hundreds of mutations. These adaptations can produce trade-offs, where fitness in the original growth conditions or resistance to other extremes is reduced (Caspeta and Nielsen, 2015; Cheung et al., 2021). Alternating selective conditions, such as switching between high and low temperature between growths, can produce different adaptations than selection in constant conditions (Lambros et al., 2021; Carpenter et al., 2023). Some trade-offs may be a reflection of the chosen selective conditions, not an underlying innate limit of biology.

**Figure 3:**
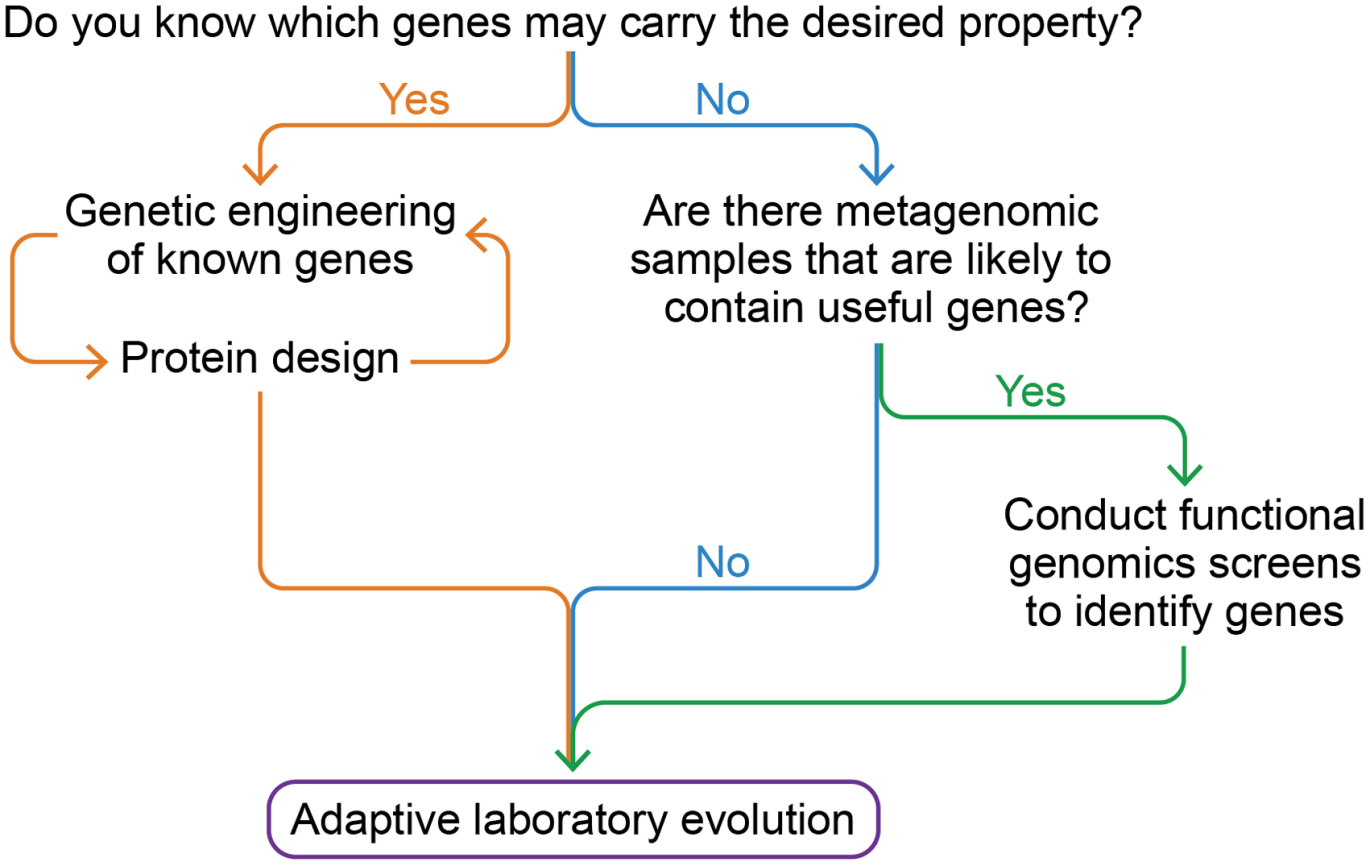
Polyextremophiles bioengineering approaches. This graph sketches the current approaches taken to evolve microorganisms, which can be combined in series to maximize the resilience achieved.

Extremophile engineering can combine strategies logically (Figure 3). If there are known genes with known functions conferring resistance to the target extreme condition, they can be used as a starting point for engineering and directed evolution. If no usable sequence-function relationships are known, metagenomic screens and functional genomics can discover new genes with the desired function (Mirete et al., 2016). Adaptive laboratory evolution can be used to further integrate, refine and evolve the transferred genes within the original genome, adjusting molecular interactions, protein stability, expression levels, burden, etc.

## Conclusion

Our consistently improving understanding of extremophiles and their mechanisms of adaptation, together, provide new opportunities to actively engineer new extremophilic capabilities. Today, extremophile properties that are simple to simulate in the lab (especially high temperature tolerance, radiation tolerance, and salt tolerance) are thoroughly studied, well mechanistically understood, and the ability to deliberately engineer these properties in target microbes has been explored. To expand our capacity to engineer biology, scientists need new tools to identify and culture unusual extremophile microbes under stringent growth conditions, such as high pressure or low gravity. Climate scientists and the biomanufacturing industry can provide the essential insights to identify the best opportunities for the engineering of extreme biology. As the field shifts to focus toward bioengineering, so too will the vocabulary — the reporting for studies must focus more directly on measuring rates of biomass or protein production under extreme conditions, rather than simply reporting binary survival or death.

Unanswered questions remain about the extent to which extremophile properties can be combined, enhanced, or transferred to new microbes. Today, we know enough about the root causes of stress under extreme conditions to deliberately equip microbes with adaptations that are likely to enhance performance in a new extreme environment. However, natural organisms continue to surprise us with new mechanisms for adaptation, making it valuable to continue to sample and study new wildtype organisms from extreme environments. Further study into the fundamental limits of life, and new methods for systematic probes of these limits, will allow us to engineer custom microbes designed to thrive in the exotic, artificial niches encountered in the future.

## Author contributions

JCA: Writing – original draft, Writing – review & editing. JM: Writing – review & editing. DS: Writing – review & editing. UN: Writing – review & editing. SP: Writing – review & editing. LV: Writing – review & editing. PS: Writing – review & editing. AH: Writing – review & editing. CC: Writing – review & editing. ED: Writing – original draft, Writing – review & editing.
