# Supplementary material for: Polyextremophile engineering: a review of organisms that push the limits of life
Source: Front Microbiol. 2024 Jun 5;15:1341701. doi: 10.3389/fmicb.2024.1341701 (PMC11188471; doi:10.3389/fmicb.2024.1341701)
Supplement: Supplementary file 1 [file Data_Sheet_1.PDF]

Table S1: Limits of thriving and surviving

Sources for Figure 1. Each study quantifies the ability of organisms to thrive or survive in various extreme conditions.

Organism	Temperature (°C)	pH	Salinity (% NaCl)
Bacillus subtilis	Several strains of Bacillus subtilis, which normally are mesophilic bacteria, can be adapted to grow at elevated temperatures, as high as 72°, by slowly increasing the temperature of incubation[1].<br><br>Figure 2 The inactivation rate of B. subtilis in water (▪), the overall water temperature (○) and pH value (□) at different treatment time[2].	B. subtilis was cultured to early log phase at pH 8.5 or at pH 6.0[2].<br><br>Figure 4. Inactivation of B. subtilis in water with different preset pH values: pH = 1 (▴), pH = 2 (○) and pH = 3 (▪)[3].<br><br>Figure 2 The inactivation rate of B. subtilis in water (▪), the overall water temperature (○) and pH value (□) at different treatment time[2].	Minimum inhibitory was observed at different salt concentrations that were from 0% to 10% salt of NaCl. Bacteria (Bacillus subtilis NA2) showed tolerance up to 8% against salt. Bacterial growth was observed at 0 to 8%, and then it started to decline as clearly shown in Figure 2[4]<br><br>Table 2[5]
Deinococcus radiodurans	A heat shock at 42°C maximized survival at the lethal temperature of 52°C and a cold shock at 20°C maximized survival after repeated freeze-thawing[6]	Supporting doubling times of 3.5–3.7 h in a pH range of 6.4–7.0[7].	The effect of NaCl treatment on the growth of D. radiodurans was investigated to determine the concentration of NaCl to be used in the microarray analysis. Exponentially growing D. radiodurans cultures were challenged with 0.1 M, 0.3 M, 0.5 M, and 2 M NaCl. The growth of D. radiodurans was gradually retarded with increasing concentrations of NaCl and was almost completely inhibited by the addition of 2 M NaCl (Fig. 1)[8]
Thermus aquaticus	Figure 1[9]	Page 295[9]	T. aquaticus is very sensitive to changes in salinity. Its growth is inhibited even if there is a 0.5% concentration of NaCl and cannot grow in any system with NaCl concentrations above 1% [10].
Psycromonas ingrahaii	*P. ingrahamii* is notable in that it grows at a temperature of -12 degrees C with a generation time of 240 h[11]	Growth was observed at near neutral pH values (pH 6.5, 6.8 and 7.4), but not at moderately acidic (pH 5.0) or basic (pH 8.3, 9.0) values[12].	Requirement for and tolerance to NaCl were determined by observing growth on CLED agar (Difco) supplemented with 0–22% NaCl. Strain 37T required NaCl for growth, showing no growth at 0% NaCl. It grew well at 1–12% NaCl, and weak growth was observed at NaCl concentrations as high as 20%[12].
Thermus thermophilus	This strain is able to grow between 55 and 80 °C with a pH range of 6–10[13,14].	-	Figure 3[15].

1. Dowben, R. M. & Weidenbüller, R. Adaptation of mesophilic bacteria to growth at elevated temperatures. *Biochim. Biophys. Acta* **158**, 255–261 (1968).
2. Wilks, J. C. *et al.* Acid and base stress and transcriptomic responses in Bacillus subtilis. *Appl. Environ. Microbiol.* **75**, 981–990 (2009).

3. Sun, P. *et al.* Inactivation ofBacillus subtilisSpores in water by a direct-current, cold atmospheric-pressure air plasma microjet. *Plasma Process. Polym.* **9**, 157–164 (2012).
4. Gul, S. *et al.* Application of Bacillus subtilis for the Alleviation of Salinity Stress in Different Cultivars of Wheat (Tritium aestivum L.). *Agronomy* **13**, 437 (2023).
5. Nagler, K., Setlow, P., Reineke, K., Driks, A. & Moeller, R. Involvement of Coat Proteins in Bacillus subtilis Spore Germination in High-Salinity Environments. *Appl. Environ. Microbiol.* **81**, 6725–6735 (2015).
6. Airo, A., Chan, S. L., Martinez, Z., Platt, M. O. & Trent, J. D. Heat shock and cold shock in Deinococcus radiodurans. *Cell Biochem. Biophys.* **40**, 277–288 (2004).
7. Holland, A. D., Rothfuss, H. M. & Lidstrom, M. E. Development of a defined medium supporting rapid growth for Deinococcus radiodurans and analysis of metabolic capacities. *Appl. Microbiol. Biotechnol.* **72**, 1074–1082 (2006).
8. Im, S., Joe, M., Kim, D., Park, D.-H. & Lim, S. Transcriptome analysis of salt-stressed Deinococcus radiodurans and characterization of salt-sensitive mutants. *Res. Microbiol.* **164**, 923–932 (2013).
9. Brock, T. D. & Freeze, H. Thermus aquaticus gen. n. and sp. n., a nonsporulating extreme thermophile. *J. Bacteriol.* **98**, 289–297 (1969).
10. Prokaryotes, P. *Thermus Species*. (Springer, New York, NY, 2012). doi:10.1007/978-1-4615-1831-0.
11. Breezee, J., Cady, N. & Staley, J. T. Subfreezing growth of the sea ice bacterium ‘Psychromonas ingrahamii’. *Microb. Ecol.* **47**, 300–304 (2004).
12. Auman, A. J., Breezee, J. L., Gosink, J. J., Kämpfer, P. & Staley, J. T. Psychromonas ingrahamii sp. nov., a novel gas vacuolate, psychrophilic bacterium isolated from Arctic polar sea ice. *Int. J. Syst. Evol. Microbiol.* **56**, 1001–1007 (2006).
13. Valenzuela, B., Solís-Cornejo, F., Araya, R. & Zamorano, P. Isolation and Characterization of Thermus thermophilus Strain ET-1: An Extremely Thermophilic Bacterium with Extracellular Thermostable Proteolytic Activity Isolated from El Tatio Geothermal Field, Antofagasta, Chile. *Int. J. Mol. Sci.* **24**, 14512 (2023).
14. Sonnleitner, B., Cometta, S. & Fiechter, A. Growth kinetics of Thermus thermophilus. *European journal of applied microbiology and biotechnology* **15**, 75–82 (1982).
15. Silva, Z. *et al.* Osmotic adaptation of Thermus thermophilus RQ-1: lesson from a mutant deficient in

synthesis of trehalose. *J. Bacteriol.* **185**, 5943–5952 (2003).
